# Mechanistic Insights into Neurotoxicity Induced by Anesthetics in the Developing Brain

**DOI:** 10.3390/ijms13066772

**Published:** 2012-06-04

**Authors:** Xi Lei, Qihao Guo, Jun Zhang

**Affiliations:** 1 Department of Anesthesiology, Huashan Hospital, Fudan University, Shanghai 200040, China; E-Mail: anesthesia2006xi@163.com; 2 Department of Neurology, Huashan Hospital, Fudan University, Shanghai 200040, China; E-Mail: dr.guoqihao@126.com

**Keywords:** general anesthetics, apoptosis, neurotoxicity, mechanisms, developing brain

## Abstract

Compelling evidence has shown that exposure to anesthetics used in the clinic can cause neurodegeneration in the mammalian developing brain, but the basis of this is not clear. Neurotoxicity induced by exposure to anesthestics in early life involves neuroapoptosis and impairment of neurodevelopmental processes such as neurogenesis, synaptogenesis and immature glial development. These effects may subsequently contribute to behavior abnormalities in later life. In this paper, we reviewed the possible mechanisms of anesthetic-induced neurotoxicity based on new *in vitro* and *in vivo* findings. Also, we discussed ways to protect against anesthetic-induced neurotoxicity and their implications for exploring cellular and molecular mechanisms of neuroprotection. These findings help in improving our understanding of developmental neurotoxicology and in avoiding adverse neurological outcomes in anesthesia practice.

## 1. Introduction

Perinatal life and early childhood are the most intensive periods of brain development, during which the fetus, infants and children undergo an eruption in neuronal proliferation, differentiation, synaptogenesis, and rapid development of dendrites to establish the complicated networks of the central nervous system. However, environmental stresses can greatly impair brain development, not only before but also after birth. For example, children who require surgical interventions, are exposed to many stressors including mental, pain, inflammatory and anesthesia, which could affect brain and behavioral development. Evidence is mounting that anesthetic exposure leads to a number of molecular, cellular and behavioral changes in the developing brain, and these effects can be harmful and long-lasting. Although other factors mentioned above may also contribute to developmental neurotoxicity, it is not discussed in this review.

Since Jevtovic-Todorovic *et al*. first put forward that anesthesia could cause widespread apoptosis and neuronal degeneration in the immature animal brain [1], there has been increased interest on how anesthetics might influence brain development in humans. Although causal correlation between anesthesia and neuronal cell death has not been established and large clinical trials are still awaiting definitive results [2], there are several human cohort studies that suggest an association between early exposure to anesthetics and poor cognitive performance in later life [3–14]. Data from animal experiments have strongly indicated that anesthetics commonly used in the clinic can induce neuronal apoptosis and impairment of normal synapse development and conformation. These effects can later result in substantial brain and behavioral abnormalities in mammals in rats, mice, guinea pigs [15], and nonhuman primates [16,17]. Such neurotoxicity has been observed for both inhalational anesthetics (N_2_O [18], isoflurane [19,20], sevoflurane [21,22], and deflurane [23]) and intravenous anesthetics (ketamine [24–26], propofol [27], thiopental [28], diazepam [29]) used alone or in combination. These findings cannot be translated to pediatric and obstetrics anesthesia practice for now, but they may provide insights into underlying mechanisms on neurotoxicity induced by anesthetics in the developing brain. More targeted and focused clinical research is needed to achieve this. Currently, the mechanisms underlying anesthetic-induced neurotoxicity are not fully understood, and it is prudent to investigate these mechanisms as exposure to general anesthesia may be putting our pediatric patients at risk.

## 2. Anesthetic Exposure and Timing

### 2.1. Exposure Concentration and Duration

The type of anesthetic, exposure concentration and duration vary among the anesthetic-induced neurotoxicity studies. For example, 4–6 h of exposure is usually selected in *in vitro* and *in vivo* studies based on the assumption that a high concentration of anesthetic or long duration of exposure results in significant neurotoxicity. Indeed, a lower volatile anesthetic concentration (for example, 0.5 minimum alveolar concentration (MAC)) is usually used to protect against ischemic brain injury, and does not result in significant cytotoxicity. This is not always the case, however, as hippocampal caspase-3 mRNA levels, an indicator of cell death, begin to increase significantly in isoflurane-treated developing rat hippocampal neurons after 6 h of exposure to 0.25 MAC isoflurane [30].

The anesthetics administered to the developing brain differ in their neurotoxic profiles. Early studies have shown that chronic exposure (4 h every day for 30 days) to subanesthetic concentration of halothane, sevoflurane and desflurane (0.1%, 0.3%, and 0.6% in 3 L/min O_2_, respectively) impair behavioral functions in adult rats [31]. However, alterations in learning and memory functions are greater with desflurane than with halothane and sevoflurane. Consistent with this result, Kodama *et al*. demonstrated that exposure to desflurane (8%) induces more neuroapoptosis than exposure to equivalent doses of isoflurane (2%) and sevoflurane (3%) in neonatal mice [32]. Results from Istaphanous *et al*. have shown that equipotent anesthetic concentrations (0.6 MAC) of desflurane, isoflurane, or sevoflurane have similar neurotoxic effects on the cortex of neonatal mice [33]. However, desflurane but not isoflurane treatment induces almost no apoptosis or neurocognitive dysfunction in cultured mouse hippocampus neurons, mouse hippocampus, and mice [34]. This difference between the two volatile anesthetics may be due to a difference in the effects of these anesthetics on mitochondrial function. This inconsistency is unclear, and may also result from differences in animal models and experimental conditions. Even so, exposure to a combination of anesthetics (for example, nitrous oxide and desflurane) may cause more severe neuroapoptosis than to a single agent by itself, which suggests a positive correlation between increased levels of anesthesia and increased severity of neurotoxicity.

### 2.2. Age Dependency of Apoptotic Neurodegeneration

The susceptibility of the developing brain to anesthetic-induced neurotoxicity compared to the mature brain has long been recognized. Stratmann *et al*. recently demonstrated that isoflurane treatment decreases progenitor proliferation in the dentate gyrus of postnatal days (PND) 7 rats and induces long-term neurocognitive dysfunction, whereas PND 60 rats are unaffected [35]. Their findings were confirmed by Zhu *et al*. [36]. These results suggest that the developing brain is more vulnerable than adult brain to effects from general anesthesia administration. In the rodent brain, evidence suggests that this type of injury is age-dependent, peaking at P7, diminishing by P14, and absent by P21 [37]. The vulnerability of the developing brain is dependent on two main exposure issues. The first factor relates to whether an agent or its active metabolite(s) reaches the developing nervous system, and the second factor relates to the period of exposure. Exposure to environmental toxins coincident with the ontogeny of neural developmental processes is more likely to cause adverse effects if the toxin exposure interferes with the cascade of neurodevelopmental processes. Obviously, general anesthetics used in pediatric or obstetric medicine meet these two criteria. Anesthetic effects on the brain during its growth spurt period have led us to recognize that a developmental insult can initiate a cascade of alterations in neurodevelopment which can be detected structurally or functionally. Furthermore, exposure during development may have adverse effects that manifest later.

### 2.3. Nonhuman Primate Studies

Anesthetic-induced neurotoxicity studies on nonhuman primates may have more clinical relevance to human patients. In primate neonatal brains, apoptotic and necrotic neuronal damage is apparent when N_2_O (70%) is combined with isoflurane (1%) treatment for 8 h in the frontal cortex, temporal gyrus and hippocampus. Furthermore, electron micrographs indicate typical swelling of the cytoplasm and nuclear condensation in the frontal cortex. These data suggest that prolonged exposure to inhaled anesthetics (a combination of N_2_O and isoflurane) in the developing monkey results in neuronal damage [17]. In another study, however, Brambrink *et al*. demonstrated that treatment for 5 h with volatile anesthetic isoflurane (0.5–1.5% end tidal concentration) alone induced a cerebral cortex neuroapoptosis response in the 6-day-old rhesus macaque brain [38]. Slikker and colleagues [39] reported that infusion of intravenous anesthetic ketamine for 9 or 24 h triggers neuroapoptosis in the rhesus macaque brain at PND 5. In addition, it was shown that a 24 h ketamine infusion triggers neuroapoptosis in the gestational day 120 (full term = 165 days) fetal rhesus macaque brain [40]. Furthermore, a shorter period of exposure (5 h) to ketamine infusion (necessary to achieve the desired anesthetic depth) also induces a significant and widespread increase in caspase-3 activation in both fetal and neonatal rhesus macaque brains [41]. However, the pattern of neurodegeneration in fetuses is different from that in neonates, and loss of neurons attributable to ketamine exposure is 2.2 times greater in the fetal than neonatal brains. Another major difference between the fetal and neonatal response to ketamine is that many of the caudal and subcortical brain regions are affected preferentially in the fetal brains, including the cerebellum and brainstem, while little or no neuroapoptotic response is observed in the neonatal brains. The pattern and density of neuroapoptosis in several brain regions after isoflurane *versus* ketamine exposure are also different.

### 2.4. Clinical Evidence and Implication

The experimental findings showing that commonly used anesthetics can induce neurotoxicity in the developing brain of animal models provoke great concerns regarding the safety of anesthesia for infants and children. Several retrospective cohort studies have indicated that early exposure to general anesthesia/surgery may place pediatric patients at risk for later learning and behavior impairment [4,5,8,13]. Those children receiving anesthesia before 3 years of age are more likely to have learning and behavior disorders compared with peers without anesthesia [7,13]. It seems that exposure to anesthesia in early life more than once or for a prolonged period adversely affects long-term neurodevelopmental outcomes in children [6,42]. This is consistent with studies in which neonatal animals (at the synaptogenesis period) exposed to anesthetics develop abnormal cognition and behavior. If neurocognitive dysfunction caused by general anesthesia is confirmed, then it is reasonable to speculate that neuropathological changes observed in the developing brain of animals similarly occur in brains of infants and children after anesthesia. However, coexisting conditions (low birth weight, medical problem, and especially surgical trauma) may preclude verification of the effect of anesthesia on cognitive development in human for ethical reasons.

## 3. Molecular Mechanisms: Neuroapoptosis *In Vitro* and *In Vivo*

Apoptosis can occur physiologically in the mammalian brain during the period of the growth spurt. Since disruption of physiological processes may result in neurodevelopmental disorders, it is important to develop a better understanding of mechanisms of anesthetic-induced neuroapoptosis in the developing brain.

Similar to many anesthetics used in clinical practice, ethanol has long been recognized to be neurotoxic to the developing brain. Exposure to ethanol during brain development might promote neurodevelopmental defects. Ethanol potentially damages the developing brain by affecting neurogenesis, cell migration, or cell survival via different intracellular signaling pathways in prenatal rat cortical and hippocampal neurons [43,44]. Although the underlying molecular mechanisms of ethanol neurotoxicity are not completely understood, mitochondrial dysfunction, altered calcium homeostasis and apoptosis-related proteins have been implicated. Increased cytosolic free calcium ([Ca^2+^]_i_) and lowered mitochondrial transmembrane potential after ethanol exposure significantly decrease the expression of anti-apoptotic protein (Bcl-2), increase expression of proapoptotic protein Bax, and stimulate the release of cytochrome-c from mitochondria in primary rat cortical neurons [45]. Other mechanisms include activation of microglia and astrocytes, causing the production of proinflammatory factors, and upregulation of NADPH oxidase (NOX) [46], thus leading to generation of ROS and disruption of mitochondrial membrane potential [47]. These molecular events that may underlie ethanol-induced neurotoxicity are reminiscent of that in anesthetic-induced neurotoxicity, and this similarity will be discussed in detail.

### 3.1. NMDA Receptors and GABA_A_ Receptors

Since the first report on neurotoxic effects of *N*-methyl-d-aspartate (NMDA)-receptor antagonists on rats during the early stage of central nervous system (CNS) development [48], the toxic effects of NMDA receptor antagonists on the immature brain have been extensively explored. Ethanol, in particular, has been studied for its NMDA antagonist and GABAergic properties because of the implication of the findings on human fetal alcohol syndrome.

As a non-competitive NMDA receptor antagonist, ketamine is commonly used for pediatric anesthesia and analgesia. Although clinical studies have not found any evidence for ketamine neurotoxicity in children undergoing surgery for cardiopulmonary bypass [49], recent experimental studies have reported that ketamine causes neuronal cell death in developing rodents and nonhuman primates. Multiple injections of 20 mg/kg ketamine significantly increase neuronal cell death in the frontal cortex, which exhibits an upregulation in the protein and mRNA expression of NMDA receptor NR1 subunit, while lower dose and fewer injections of ketamine did not have any significant effects in PND 7 rat pups [50]. Ketamine exposure (24 h of clinically relevant anesthesia) also causes a significant increase in NMDA receptor NR1 subunit mRNA expression and in neuronal cell death in perinatal rhesus monkeys [39]. These results suggest that ketamine may cause a compensatory up-regulation of NMDA receptors, subsequently triggering expression of apoptosis-related genes in the developing neurons.

In the past several decades, neuroscientists have realized that not only NMDA receptor antagonists but also gamma-aminobutyric acid type A (GABA_A_) receptor agonists can affect neurodevelopment. Currently, anesthetics, sedatives or anticonvulsants [51] used clinically act either as NMDA receptor antagonists or as GABA_A_ receptor agonists. Recent reports have indicated that sedative or anticonvulsant drugs (MK801, phenobarbital, and diazepam) could suppress postnatal neurogenesis [52], and exposure to general anesthetics that block NMDA receptors or potentiate GABA receptors can trigger widespread apoptotic cell death in developing rodent brain, eventually resulting in long-term neurobehavioral impairment. How anesthesia initiates the neurotoxicity mechanism involving the NMDA receptor or GABA_A_ receptor is not clear. Zhou *et al*. demonstrated that 70% nitrous oxide and 0.75% isoflurane exposure for 6 h significantly increase neuroapoptosis of glutamatergic, GABAergic and dopaminergic neurons in the developing brain but not that of the cholinergic neurons in the basal forebrain [53]. These results suggest that anesthetics displaying similar properties to that of NMDA receptor antagonist or GABA receptor agonist induce damage in a cell type-specific manner.

Contrary to previous findings that isoflurane can exert effects on the glutamatergic synapse by reducing pre-synaptic glutamate release and increasing its uptake from the synaptic cleft [54], a ^1^H NMR spectroscopy study demonstrated that recurrent 2% isoflurane anesthesia exposure during mouse development increases glutamate levels in the posterior cortex [55]. It is possible that inhibition of NMDA receptors in neurons by anesthetics can induce excessive glutamate release as a compensatory mechanism. Glutamate neurotoxicity is apparent even after anesthesia administration, and so NMDA antagonist can potentially attenuate neuroapoptosis [56]. However, it seems that isoflurane-induced neuroapoptosis could be independent of activation of the GABA_A_ receptors because the GABA_A_ receptor antagonist gabazine does not attenuate caspase-3 activation in organotypic hippocampal slice cultures [57].

### 3.2. Mitochondrial Perturbations

Mitochondria are not only critical in cell metabolism, but also play essential roles in controlling apoptosis. Injured mitochondria could be a significant source of reactive oxygen species (ROS) which, if not scavenged properly, may cause excessive lipid peroxidation and damage of cellular membranes. Yon *et al*. reported that the impairment of mitochondrial integrity is one of the first signs of neuronal dysfunction after anesthesia exposure [58]. In addition, Zhang *et al*. observed that treatment with 2% isoflurane for 6 h can increase Bax levels, decrease Bcl-2 levels, increase ROS accumulation, facilitate cytochrome c release from the mitochondria to the cytosol, induce activation of caspase-9 and caspase-3, and finally cause apoptosis *in vitro* and *in vivo* [59]. In another study, isoflurane induces neurotoxicity through opening of the mitochondrial permeability transition pore (mPTP), elevation in ROS levels, reduction in mitochondrial membrane potential and adenosine-5′-triphosphate(ATP) production, and activation of caspase-3 in cultured cells, mouse hippocampus neurons and mouse hippocampus [34]. Conversely, a blocker of mPTP opening minimizes isoflurane-induced mPTP opening, increase in ROS levels, and caspase-3 activation. These results suggest that a disturbance in mitochondrial integrity and function could be early events, and that activation of the mitochondria-dependent apoptotic pathway is one of the intrinsic mechanisms underlying isoflurane-induced neuronal damage. Sanchez *et al*. further examined the long-term effects of a commonly used anesthesia combination (isoflurane, nitrous oxide, and midazolam) on the regional distribution, ultrastructural properties, and electron transport chain function of mitochondria in the subiculum of rat pups [60]. This anesthesia causes significant enlargement and destruction of mitochondrial structure, increase in mitochondrial complex IV activity and a twofold decrease in mitochondrial regional distribution in presynaptic neuronal profiles, which may compromise the normal development and functioning of synapses. If administered during brain development, this anesthesia causes significant upregulation in ROS, accompanied by significant membrane lipid peroxidation, mitochondrial damage (swollen mitochondria with balloon-like cristae, disorganized matrices and vacuolation in early stage, disappearance of the separation between the inner and outer membranes in late stage), and neuronal loss in the subiculi of Sprague-Dawley rat pups at PND 7 [61]. Anesthesia exposure did not impair rats’ nutritional development. Administering a ROS scavenger or a mitochondria protectant around the time of anesthesia exposure results in intact mitochondrial integrity, significant downregulation of ROS and lipid peroxidation, prevention of mitochondrial morphological damage, protection of neuropil, and prevention of neuronal loss. Most importantly, peri-anesthesia treatment with an ROS scavenger or mitochondria protectant prevented anesthesia-induced cognitive impairment. These results strongly suggest that exposure to general anesthesia can impair mitochondrial morphogenesis, integrity and function at the peak of synaptogenesis, and that this mitochondrial impairment may be central in anesthetic-induced acute neuroapoptosis and cognitive abnormalities in later life.

### 3.3. Dysregulation of Intracellular Ca^2+^ Homeostasis

A previous study has shown agents such as MK801 (which blocks ligand-gated calcium entry), BAPTA (which chelates intracellular calcium), and thapsigargin (which inhibits the endoplasmic reticulum Ca^2+^-ATPase pump) could profoundly influence early neuronal maturation (growth cone expansion, neurite length, neurite complexity) in embryo day 18 rat cortical neurons [62]. Increasing intracellular Ca^2+^ using ionomycin reverses neurotoxicity [63]. This disruption in calcium homeostasis may also be toxic to developing neurons. Turner *et al*. attributed the primary event of this injury involving interference with NMDA or GABA_A_ receptor signaling to loss of calcium [64], since loss of calcium promotes several events including (1) mitochondrial dysfunction and cytochrome C release; (2) decreased calcium and increased active caspase-3; and (3) growth cone collapse, as well as reduced neurite length and complexity. Other observations implicating calcium include (1) that ketamine-like brain damage can be mimicked by calcium channel blockade or prevented by calcium channel activation; (2) MK801-induced active caspase-3 is not observed in cells expressing calcium binding proteins (CaBPs); and (3) the postnatal surge in CaBP expression overlaps well with age-dependent loss of sensitivity to NMDA receptor blockade. Downstream of loss of intracellular calcium likely involves changes in expression of proteins related to the cytoskeleton, synapse, production of neurotransmitters, or calcium buffering. Interestingly, studies have demonstrated that volatile anesthetics including isoflurane and sevoflurane could induce intracellular calcium overload, which increases ROS and NO levels that could result in neuroapoptosis. However, the mechanism of calcium overload remains unclear.

Previous studies have demonstrated that prolonged exposure of the young brain to anesthetics will increase the levels of NR1 receptor on the neuronal membrane [65], and this can promote a glutamate storm. The glutamate storm would result in the Ca^2+^ efflux in neuron. On the other hand, the primary inhibitory neurotransmitter GABA is excitatory on immature neurons via its action at the GABA_A_ receptor, where it depolarizes the postsynaptic membrane potential [66] due to the absence of expression of KCC2 K^+^/Cl^−^ cotransporter and it induces a cytosolic Ca^2+^ concentration increase. Isoflurane can enhance the GABA-induced [Ca^2+^]_i_ increase and potentiate GABA_A_ receptor-mediated synaptic voltage-dependent calcium channels (VDCCs) peak current amplitude in a dose-dependent manner in immature rat hippocampal neurons. Intracellular calcium channels blocker dantrolene and l-type calcium channel blocker nicardipine markedly inhibit this enhancement mediated by isoflurane. The [Ca^2+^]_i_ overload induced by isoflurane activates caspase-3 and increases neuronal apoptosis [30]. Wei *et al*. has ascribed this cytotoxicity induced by volatile anesthetics to increased cytosolic Ca^2+^ released from endoplasmic reticulum (ER) via activation of IP3 receptors or ryanodine receptors. Interestingly, IP_3_ is the downstream signal of the activated GABA receptor. Local Ca^2+^ influx can be triggered by intracellular stores, Ca^2+^ efflux in turn triggers Ca^2+^ release via neighboring inositol triphosphate and ryanodine receptors (Ca^2+^-induced Ca^2+^ release). These events result in the elevation of neuronal intracellular Ca^2+^ concentration even after the washout of anesthetics [67].

Ca^2+^ oscillation is the periodical increase and decrease of intracellular Ca^2+^ concentration. Ca^2+^ oscillation can interact with the neuronal nuclear transcriptional machinery [68]. In the immature brain, Ca^2+^ oscillation plays an important role in neuronal differentiation, synaptogenesis, neuronal network development and plasticity [69]; it can increase CaMK II levels which would then promote neuronal synaptic plasticity [70], and synapsin levels, improving neuronal synaptogenesis. However, a persistent intracellular Ca^2+^ concentration not only interferes with Ca^2+^ oscillation, which would affect neuronal synaptogenesis, but also leads to neuronal apoptosis. Sinner B *et al*. found that upon anesthesia exposure, the amplitude and frequency of the cytosolic Ca^2+^ oscillation are attenuated in a concentration-dependent manner in E19 old rat neurons. Concomitant to this is a decrease in CaMK II and synapsin expression [67,71,72]. Viberg *et al*. [26] found that exposure of mice at PND 10 to general anesthesia results in an increase in the levels of CaMK II and growth associated protein-43 (GAP-43) in the hippocampus. These results, though seemingly inconsistent with the results of Sinner *et al*. [67,71], highlight the age-dependent vulnerability of synaptogenesis to anesthetics. The relationship between the Ca^2+^ oscillation reduced by anesthetics and neuronal apoptosis needs further investigation.

### 3.4. Neuroinflammatory Pathway

Mounting evidence suggests that the inflammatory response may be a critical component in different brain pathologies. However, a link between neuroinflammation and anesthesia- and surgery-induced neuropathology is not fully understood. Recent findings suggest that neuroinflammatory mediators such as cytokines may be involved in a number of key steps in the pathological cascade of events leading to anesthetic-induced neuronal injury. This hypothesis is strongly supported by experimental and preclinical observations indicating that activation of the inflammatory reaction correlates with more neuronal damage.

Volatile anesthetic isoflurane increases the levels of proinflammatory cytokines including tumor necrosis factor (TNF)-α, interleukin (IL)-6, and IL-1β in brain tissues and primary neurons of mice [73]. These results suggest that isoflurane may cause neuroinflammation, potentially promoting Alzheimer’s disease (AD) neuropathogenesis. Shu *et al*. demonstrated that 70% nitrous oxide and 0.75% isoflurane treatment for 6 h can cause not only widespread apoptosis in the brain and spinal cord, but also increased expression of the pro-inflammatory cytokine, IL-1β, in the cortex of rat pups. Sevoflurane, another volatile anesthetic, increases TNFα levels, caspase activation, apoptosis, and beta-amyloid(Aβ) protein levels in the brain tissues of AD transgenic neonatal mice [74]. Surgical trauma can also increase transcription and expression levels of proinflammatory cytokines TNF-α, IL-1β and microglial activation in hippocampus, possibly contributing to hippocampal-dependent memory impairment [75,76]. Systemic inflammation in turn enhances surgery-induced cognitive dysfunction [77]. These neuroinflammatory effects of surgery and memory dysfunction are mitigated with blockade of TNF-α [75] or pretreatment with IL-1 receptor antagonist, and in IL-1 receptor knock out mice [78]. Moreover, nociceptive stimulation (e.g., formalin or surgical incision) with prolonged anesthesia exposure produces significantly more apoptosis than prolonged anesthesia alone in neonates during the synaptogenic period [79]. These results suggest that both anesthesia and surgery can induce cytokines release in the central nervous system, leading to deleterious neurodevelopmental effects. However, it remains to be determined if it is surgery, general anesthesia, or both, that play a major role in neuroinflammatory development and postoperative cognitive decline.

### 3.5. The BDNF Pathway

Neurotrophins are chemicals of central importance in the regulation of the survival, differentiation, and maintenance of function of neurons in the brain. The synthesis and secretion of neurotrophins depend on and are regulated by neuronal activity, which is itself related directly to environmental input. Recent evidence has shown that general anesthetics induce neuroapoptotic damage in the developing brain of the immature rats, at least in part via the brain-derived neurotrophic factor (BDNF)-modulated apoptotic cascade [80]. BDNF supports neuronal survival, differentiation and several forms of synaptic plasticity in the developing brain. BDNF is secreted from synaptic vesicles as a precursor or proneurotrophin (proBDNF). Then proBDNF is proteolytically cleaved by plasmin to become mature BDNF (mBDNF) in the synaptic cleft [81]. Plasmin is converted from plasminogen via the proteolysis of tissue plasminogen activator (tPA), a protease released from pre-synaptic vesicles [82]. BDNF has two types of receptor in the brain. One is the Trk receptor which is predominantly expressed in the normal condition. In the developing brain, the mature BDNF (mBDNF) is synthesized and released by neurons in an activity-dependent manner. BDNF then binds to the Trk receptor and activates the phosphorylated serine/threonine-specific protein kinase (Akt), promoting neuronal survival in the cytoplasm [83]. Another receptor P75^NTR^ binds preferentially to immature BDNF, proBDNF, which could result in reduced synaptogenesis, withdrawal of dendritic spines[84,85] and neuronal apoptosis [81].

However, volatile anesthetic isoflurane could inhibit the activity-dependent release of tPA [86]. An *in vitro* study has shown that isoflurane decreases the tPA released from mouse primary neurons which results in less proBDNF cleaved to become mBDNF. Consequently, proBDNF accumulates in the cleft and binds with the P75^NTR^ receptor, which then triggers apoptosis leading to increased caspase-3 activation and decreased dendritic spine number [87]. This adverse effect of anesthetics can be mitigated by exogenous tPA or plasmin application [88]. Further studies have found that the activated P75^NTR^ could engage the downstream effector RhoA. RhoA is a small GTPase that when activated can promote cytoskeleton depolymerization in neurons [85]. Inhibition of RhoA activation by TAT-Peo5, an intracellular inhibitor of P75^NTR^, or by downstream stabilization of actin cytoskeleton with Jasplakinolide, significantly attenuates neuronal death in mouse hippocampi [85]. Meanwhile, less binding of mBDNF to Trk receptor results in less activated Akt, which compromises neuronal survival. Lu *et al*. observed that β-estradiol, considered as a novel neuroprotectant, can up-regulate activated Akt to protect neurons from apoptosis [80]. Pearn *et al*. demonstrated that intravenous anesthetic propofol induces apoptosis in developing neurons *in vivo* and *in vitro* through P75^NTR^ and the downstream effector RhoA kinase [89].

### 3.6. Other Signal Transduction Pathways

Wang *et al*. found that anesthesia can induce the upregulation of PKCα and p-JNK, and downregulation of p-ERK and Fos protein in the hippocampus of offspring rats exposed to 1 MAC sevoflurane for 6 h at gestation but not pregestation [22]. Similarly, Straiko *et al*. observed that anesthesia suppresses phosphorylation of extracellular signal-regulated protein kinase (p-ERK). Lithium counteracts both p-ERK downregulation and neuroapoptotic action of these anesthetic drugs [90]. On the other hand, Shu *et al*. [79] showed that prolonged anesthesia (70% nitrous oxide and 0.75% isoflurane for 6 h) plus nociceptive stimuli induce more c-Fos positive neurons in the thalamus than with anesthesia or nociceptive stimuli alone in neonatal rats. Furthermore, repeated intraperitoneal injections of intravenous anesthetic propofol upregulate c-Fos expression in the hippocampal CA3 region of infant mice (5–7 days old) [91]. This implies that PKCα, JNK (jun terminal kinase), ERK and c-Fos may be involved in anesthetic-induced neurotoxicity in the developing brain at pregnancy or neonatal stage.

Jiang *et al*. demonstrated that isoflurane, in a dose-dependent manner, can up-regulate HIF-1α protein levels in rodent primary cultured neurons and in the developing rat brain. Knockdown of HIF-1α expression in cultured neurons attenuates isoflurane-induced increase in cleaved caspase-3 and poly-(ADP-ribose) polymerase (PARP), which suggests that HIF-1α may play a role in anesthetics-induced neurotoxicity under normoxic condition [92]. Some authors have also found that anesthetics-induced neuroapoptosis and cognitive dysfunction may be associated with increase in tumor suppressor P53 expression [93].

## 4. Cellular Processes in Neurodevelopment

The mammalian brain contains a population of neurons that are continuously generated from late embryogenesis through adulthood, after the generation of almost all other neuronal types. Mounting evidence has demonstrated that general anesthetics not only induce neuroapoptosis but also affect neurodevelopmental processes at the peak of synaptogenesis via certain cellular mechanisms.

### 4.1. Neurogenesis

Treatment of animals with an NMDA receptor inhibitor leads to impaired spatial learning and memory in animals, possibly through down-regulation of neurogenesis [94]. Similar to the effects of NMDA receptor inhibition on maturation and proliferation of neuronal progenitor cells [95,96], prolonged general anesthetics and sedatives treatment affect neurogenesis in an age-dependent manner [97]. Isoflurane treatment causes loss of neural stem cells and reduced neurogenesis in neonates but not adult animals [36]. In support of this, Erasso *et al*. showed that propofol or isoflurane decreases hippocampal cell proliferation in young, but not aged rats [98]. Stratmann *et al*. did not detect any differences in hippocampal progenitor proliferation, neuronal differentiation, new neuronal survival after isoflurane administration in aged rats [99]. Interestingly, another study have found that isoflurane decreases dentate gyrus progenitor cells proliferation until at least 5 days after anesthesia in young rats, but it increases neural progenitor proliferation 5–10 days after anesthesia in adult rats [35]. Propofol [100] and S (+) ketamine [101] by itself has no impact on basal neurogenesis in adult rats, although neurogenesis is significantly increased during cerebral ischemia. *In vitro* studies have further confirmed the effects of anesthetics on neurogenesis and cell proliferations. Sall *et al*. observed that isoflurane exposure inhibits proliferation and differentiation, and increases neuronal but not glial fate selection in cultured hippocampal neural precursor cells [102]. Isoflurane at and above 1 MAC inhibits proliferation (by 20–30%) but not cell death of rat embryonic neural stem cells [103]. Interestingly, inflammatory cytokines induced by general anesthetics may also impair neural progenitor cells proliferation and alter their differentiation [104]. These results suggest that anesthetics can reduce the pool of neural stem cells and decrease their self-renewal capacity *in vivo* in an age-dependent manner, such as in postnatal hippocampal cell proliferation. These changes could adversely result in late cognitive dysfunction after general anesthesia.

### 4.2. Dendritic Development

The dendritic spines are the postsynaptic sites of most excitatory axodendritic synapses in the brain, and genesis of dendritic filopodia and spines formation play a critical role in synaptogenesis [105]. Impairment of synaptogenesis potentially interferes with the development of neural networks. Recent studies from fixed brain preparations have shown that exposure to ketamine [106] and isoflurane [87] decreases synapse or spine density in hippocampus of neonatal rodents at PNDs 5–13. However, it is unclear whether this effect of anesthetics in early postnatal development is transient or long-lasting. On the other hand, exposure to anesthetics midazolam, propofol, or ketamine treatment for 5 h, administered intravenously, causes a significant increase in the density of dendritic spines in the mouse somatosensory cortex and hippocampus at PND 15 and 20 but not PND 30 [107]. Furthermore, a substantial increase in dendritic spine density is observed in rat medial prefrontal cortex after exposure to volatile anesthetics isoflurane, sevoflurane, or desflurane for 30–120 min at PND 16 [108]. Using transgenic mice expressing yellow fluorescence protein in layer 5 pyramidal neurons, Yang *et al*. have found that ketamine-xylazine increases dendritic filopodial (spine precursors) formation whereas isoflurane decreases filopodial elimination in the mouse cortex at PND 30 during 4 h of anesthesia. However, both anesthetics have no significant effects on the number of dendritic spines, and the changes in filopodial dynamics seem transient and not long-lasting [109]. Similar to the changes observed in dendritic spines and filopodia-like structures during blocked synaptic transmission [110], these findings suggest that the effects of anesthetic exposure on synaptic connectivity in the brain may depend on developmental stage level [111]. These studies also show that the fate of synaptogenesis depends on the dose of anesthetics. A low concentration of propofol [112] or ketamine [113,114] results in a persistent decrease in dendritic growth and arbor expansion, while a high concentration directly induces neuroapoptosis in developing GABAergic interneurons in culture. The mechanisms underlying the effects on anesthetics on synaptogenesis remain unclear, but at least may, in part, involve blockade of NMDA receptor activity or potentiation of GABA_A_ receptor activity.

### 4.3. Neurite Outgrowth

In cultured Lymnaea neurons, prolonged propofol treatment severely compromises the formation of both chemical and electrical synapses, although this synaptic connection can reform between neurons several days after drug washout [115]. Propofol also decreases neuronal activity in a concentration- and time-dependent manner by causing retraction of primary cultured rat cortical neuronal neurites, and this retraction may be through an actin-myosin contraction mediated by activation of GABA_A_ receptors [116]. However, long-term treatment of cultured Lymnaea neurons with sevoflurane and isoflurane does not affect neurite regeneration and incidence of synapse formation [117]. Therefore the effects of anesthetics on neurite outgrowth are reversible and transient. This makes anesthetics unlikely to induce cognitive dysfunction by this mechanism.

### 4.4. Glial Development

Astrocytes, the most abundant glial cells in brain, are necessary for the formation, function, stability and plasticity of synapses [118]. Recently, Dallasen *et al*. have demonstrated that 2% isoflurane exposure for 2 h causes a reduction in astroglial processes in the hippocampus and dentate gyrus of adult young mice [119]. Lunardi *et al*. found that anesthesia is toxic to developing astroglia because it impairs proper cytoskeletal development early, thereby disturbing glial growth and maturation [120]. They found that anesthetics exposure results in RhoA activation, reduced levels of both myosin light chain protein (MLC), and phosphorylation of myosin light chain protein (MLC-P) in glial cells. Subsequently, anesthetics may interfere at multiple levels to impair the morphology and other aspects of glial cells. Interestingly, the susceptibility of glial cells to anesthetic toxicity is age-dependent as well. A study has shown that anesthesia impairs the growth of very immature but not more mature cultured astroglia [120]. Similar to findings in neural stem cells [102], Lunardi *et al*. did not find significant apoptosis of astrocytes *in vitro* at the dose which could cause neuronal apoptosis *in vitro* [120]. Although the mechanism behind this is not clear, it might involve a difference in the threshold of cellular apoptosis induction to anesthetic dose. Perhaps, the lethal anesthetic dose for immature glial cells and neural stem cells is greater than that for developing neurons.

## 5. Neurobehavior Mechanisms

Rapid brain development affects cognitive, social and emotional growth during the first three years of a child’s life. Such development helps to ensure that each child reaches his or her potential early, and that he/she is a productive part of a rapidly changing, global society. Brain development and later neurobehavior health are influenced by adequate nutrition, external environment, and exposure to compounds. Experimental studies have shown that learning and memory are impaired in animals exposed to general anesthesia at early life, based on results from neurocognitive tests including water maze test, fear conditioning test, and other memory tests. Most of these studies attributes this cognitive dysfunction to anesthetic-induced neuroapoptosis and impaired neurogenesis because reduction in anesthesia-induced neuroapoptosis can reverse these adverse behavior effects. However, does anesthesia-induced neuroapoptosis or neurogenesis impairment necessarily lead to cognitive, social and behavioral abnormalities in human adulthood? Recently Goldberg *et al*. [121] have proposed that a change in brain function is only associated with anesthetic-induced neurotoxicity to fast-spiking (FS) cells. These specific neuronal cells mediate feed-forward inhibition and temporal sculpting of information transfer in neural circuits, maintain excitation/inhibition balance, and contribute to network oscillations. The causal correlation between anesthetic-induced neurotoxicity and fast-spiking cells impairment remains to be established. Additionally, it is believed that the effects of volatile anesthetics on production or oligomerization of Aβ, a biomarker of AD, primarily occurs in cells with AD features or in transgenic AD mice [74]. However, human neurobehavior is undoubtedly complex, such that subtle impairments in neurobehavior resulting from anesthesia are not easily detected through current neuropsychological and neurobehavior tests. Mounting evidence has also indicated other sources of stress (surgical trauma, pain *etc*.) during the perioperative period can increase the risk of stress-related neurocognitive problems well into the adult years [122]. This makes it difficult to evaluate the relationship between anesthesia and neurotoxicity in humans. Another question is whether anesthetic-induced neurotoxic effects can be compensated during growth and differentiation of the nervous system, and that neurobehavior disorders can be restored in later life. At least one paper has demonstrated that enriched environment could reverse sevoflurane-induced long-term memory impairment in neonatal rats by restoring the survival and differentiation of newborn cells in the hippocampus [123]. The mechanisms of neurobehavioral abnormalities induced by anesthetic exposure in early life need further investigation as the findings will have a profound implication on clinical practice.

## 6. Neuroprotection against Anesthetic-Induced Neurodegeneration

The experimental data reviewed here demonstrate that general anesthetics may cause neuronal apoptosis and alter normal brain development. These findings raise concerns regarding current anesthesia practice for pregnant women, infants and children. Accordingly, it is essential to develop and explore clinically relevant neuroprotective strategies in animals.

### 6.1. Erythropoietin (EPO)

Several studies have verified that Epo and Epo receptor (EpoR) are expressed in the brain during fetal and adult life. Expression of Epo and EpoR changes significantly during brain development. *In vitro* and *in vivo* studies have shown that Epo could have a direct neurotrophic and neuroprotective effect, particularly in conditions of neural damage, such as hypoxia, ischaemia or brain haemorrhage. Moreover, it has been shown that Epo could influence the release of neurotransmitters, playing an important role in synaptic plasticity. Konishi *et al*. [124] demonstrated that Epo acts as a neurotrophic factor on central cholinergic neurons, influencing their differentiation and regeneration. Therefore Tsuchimoto *et al*. examined whether EPO could attenuate neurodegeneration induced by isoflurane in PND 7 mice [125], and found that degenerative neuronal change and learning disability in PND 7 mice are attenuated when 50,000 IU/kg rEPO is administered subcutaneously prior to a 6 h exposure isoflurane (1.0%) compared with isoflurane treatment alone. This result suggests that anesthetics may inhibit EPO production, resulting in neurotoxicity in young rodents. Interestingly, isoflurane, in a concentration-dependent manner, suppresses EPO mRNA expression in the mouse brain and in primary cultured astrocytes under hypoxia condition [126]. The neuroprotective function of EPO against anesthetic-induced neurotoxicity remains to be established.

### 6.2. Brain Preconditioning with Anesthetics

Interestingly, anesthetics that induce neurotoxicity can also deliver neuroprotection against anesthetic-induced neurotoxicity. For example, isoflurane preconditioning can protect from isoflurane-induced neurotoxicity [127]. 5 mg/kg ketamine administration at PND 6 before injection of 20 mg/kg ketamine at PND 7 in neonatal rats significantly attenuates apoptosis from 20 mg/kg ketamine-induced increase in activated caspase-3 in some brain regions, such as the retrosplenial cortex. Anesthetics, in general, can have dual effects on the brain depending on context. Similar to isoflurane, pretreatment with inertia gas anesthetic xenon can also attenuate anesthetic (70% nitrous oxide and 0.75% isoflurane)-induced cortical and hippocampal neuroapoptosis, and cognitive deterioration in the neonatal rat [93]. In line with this, Cattano *et al*. verified that xenon protected against neuroapoptosis induced by isoflurane alone in the developing mouse brain. Xenon’s neuroprotective effect may be through its ability to inhibit intrinsic and common apoptotic pathways [128]. However, results from Ma *et al*. demonstrate that xenon alone could induce a significant increase in neuroapoptosis [129]. Intravenous anesthetic propofol has similar effects on isoflurane-induced neurotoxicity. Zhang *et al*. demonstrated that propofol pretreatment can mitigate caspase-3 activation and Aβ42 oligomerization in naïve and amyloid precursor protein stably-transfected H4 human neuroglioma cells. On the other hand, the anesthetic nitrous oxide or hypoxia pretreatment cannot protect from anesthetic-induced neuroapoptosis and cognitive function impairment [93]. These data suggest that prior exposure to low dose of anesthesia, or a shorter duration of anesthetic exposure, can attenuate injury from high dose or prolonged anesthetic exposure in the developing brain. Anesthetics may then be used against themselves to prevent anesthesia-induced neuroapoptosis. These results also imply that neuroprotective mechanisms initiated by anesthetics, such as the HIF-1α or Akt pathways, may potentially become neurotoxic under prolonged anesthetic exposure, particularly in the developing brain.

### 6.3. Vitamins

Nicotinamide, a water-soluble vitamin, is a coenzyme in a wide variety of enzymatic oxidation-reduction reactions. It protects against ethanol-induced apoptotic neurodegeneration in the developing mouse brain [130]. Ullah *et al*. [131] have recently shown that a single dose of 1 mg/kg nicotinamide attenuates ketamine-induced neuronal cell loss in the developing rat brain. This reduced neuroapoptosis involves downregulation of Bax, inhibition of cytochrome c release from mitochondria into cytosol, and reduction in activated caspase-3 levels. Nicotinamide is also a potent inhibitor of proinflammatory cytokines. It may inhibit isoflurane-induced increase in levels of proinflammatarory factors TNFα, IL-6, and IL-1β, thus protecting from neurodevelopmental disorders.

Vitamin D_3_ (1-α-2,5-dihydroxy-vitamin D_3_) can also protect against ketamine-induced neuroapoptosis. Pretreatment of PND 6 animals with 20 mg/kg vitamin D_3_ prevents ketamine-induced robust apoptosis (greater than 50% reduction in activated caspase-3 levels) in somatosensory cortex at PND 7 [64]. Previous studies have shown that neuronal apoptosis due to NMDA receptor blockade does not occur in cells that express calcium binding proteins (CaBPs) [132]. Apoptosis from NMDA receptor blockade diminishes rapidly between PND 10–14, when CaBP expression rapidly increases. Vitamin D_3_ can induce CaBP expression or enhance trophic factor action, both of which can stabilize intracellular calcium.

Vitamin C, known as an antioxidant, can eliminate oxidative stress and has been used to treat ethanol-induced neurotoxicity [47,133]. Therefore, it may also be effective against anesthetic-induced neurotoxicity. Since these vitamins are already in clinical use, investigating its neuroprotective role has high translational value.

### 6.4. Alpha2(α_2_) Adrenoceptor Agonist

Dexmedetomidine produces sedation and analgesia without respiratory depression by activating central α_2_ adrenoceptors. As α_2_ adrenoceptor signaling plays a trophic role during neurodevelopment and is neuroprotective in several settings of neuronal injury, Sanders *et al*. have examined whether dexmedetomidine can protect against isoflurane-induced neurotoxicity [57]. They found that dexmedetomidine, in a dose-dependent manner, can prevent against isoflurane-induced injury in the hippocampus, thalamus, and cortex, and long-term memory impairment in neonatal rats. This neuroprotection is attenuated by an α_2_ adrenoceptor inhibitor. Dexmedetomidine neuroprotection appears to involve a decrease in cleaved caspase-3 levels, and reversal of isoflurane-induced decrease in anti-apoptotic Bcl-1, pERK1, and pERK2 protein expression *in vivo* [57]. These results suggest that α_2_ adrenoceptor signaling activated by dexmedetomidine antagonizes apoptosis due to its neurotrophic effect, subsequently reducing caspase-3 activation in the developing brain [134]. Neuroprotective mechanisms of α_2_ adrenoceptor signaling also involve inhibition of calcium entry, scavenging of glutamate, and reduction in NMDA receptor activation [135]. However, interestingly, another α_2_ adrenoceptor agonist clonidine has been reported to increase caspase-3 mRNA levels and DNA fragmentation in the developing rat brainstem but not cortex [136], and this may induce long-lasting alterations in brain neurochemistry, autonomic functions, and behavior.

### 6.5. Lithium

Recently, Liu *et al*. have demonstrated that volatile anesthetic sevoflurane (2% inspired) treatment induces significant impairment of retention performance on a 24 h learning test, and inhibits phosphorylation of glycogen synthase kinase-3β (GSK-3β) in the hippocampus of rats 2 h after inhibitory avoidance retention training. Lithium chloride pretreatment (100 mg/kg, intraperitoneally) not only blocks sevoflurane-induced impairment of memory consolidation, but also reverses the inhibitory effect of sevoflurane on GSK-3β phosphorylation in the hippocampus [137]. This makes lithium a promising drug in the prevention of anesthetic-induced neurotoxicity. In fact, lithium, as a GSK-3β inhibitor, has shown protective effects against neuroapoptosis induced by drugs or hypoxic-ischemic brain injury in the developing brain [138]. Mounting evidence has indicated that lithium could be used for preventing postoperative spatial learning and memory deficits in animals [139,140]. This protective effect of lithium may be through inhibition of hippocampal GSK-3β activation or enhancement of GSK-3β phosphorylation state. Lithium treatment can also significantly increase BDNF serum levels, and suppress neuroapoptosis in central nervous system through the BDNF-Akt-Bcl2 antiapoptotic signaling pathway [141].

### 6.6. Activity-Dependent Neuroprotective Protein (ADNP)

More recently, activity-dependent neuroprotective protein (ADNP) (or its fragment peptide NAPVSIPQ [NAP]) has shown great promise against hypoxic-ischemia brain injury of neonatal rats [142]. The microarray analysis of the somatosensory cortex from ketamine-treated PND7 rat pups has revealed that expression of activity-dependent neuroprotective protein (ADNP) is enhanced. Injection of NAP 15 min before ketamine administration could diminish ketamine-induced activated caspase-3 in somatosensory cortex in a dose-dependent manner, and at the 20 mg/kg dose, caspase-3 levels match that of vehicle controls [64]. Activity-dependent neurotrophic factor (ADNF) may have similar neuroprotective effects on NMDA receptor inhibitor-induced neurotoxicity.

### 6.7. Other Neuroprotectants

Other neuroprotectants are also reported to alleviate anesthetic-induced neurotoxicity in the developing brain. Melatonin, a sleep-promoting agent and antioxidant, can protect impairment of cerebral cortex and anterior thalamus from an anesthesia cocktail (midazolam, isoflurane, nitrous oxide) in rat developing brain [143], similar to what has been observed in neonatal hypoxic-ischemic rat model [144]. This neuroprotective effect may be mediated by inhibition of mitochondria-dependent apoptotic pathway. Acetyl-l-carnitine, another neuroprotectant, is metabolized in the brain to acetyl coenzyme A which subsequently enters the tricarboxylic acid cycle. It has been found to effectively block neuronal apoptosis caused by exposure to a combination of N_2_O and isoflurane for 6 h or more [145], or by traumatic brain injury [146] in the developing rat brain. Both EUK-134, a synthetic ROS scavenger, and R (+) pramipexole (PPX), a synthetic aminobenzothiazol derivative that restores mitochondrial integrity, can inhibit ROS upregulation, lipid peroxidation, and mitochondrial injury in the subiculum of rat after general anesthesia, thereby preventing anesthesia-induced cognitive impairment [147]. In addition, overexpression of heat shock protein 72 which can confer mitochondrial protection, also prevents early postoperative memory deficit induced by anesthesia and/or surgery [148]. These results suggest that the mitochondria may be central to anesthetic-induced neurotoxicity, as agents which can maintain mitochondrial function, suppress ROS release and promote bioenergy production have a neuroprotective effect in animal models of anesthetic-induced neurotoxicity [149].

## 7. Summary

In spite of inconsistent findings [150], mounting evidence from rodents to primates suggests that anesthetics exposure of the developing mammalian brain during the critical stage of synaptogenesis results in increased neuroapoptosis and associated long-term neurocognitive deficits. Anesthetics may potentially induce neurodevelopmental defects much earlier than this period [151]. Moreover, nociceptive stimuli such as surgery can enhance prolonged anesthesia-induced neuroapoptosis in the rat developing brain [79]. Although there is a dispute on whether anesthesia can cause long-term neurological consequences in surgical patients, a recent epidemiologic study [152] and a case report [153] have indicated a potential association between anesthesia/surgery and subsequent cognitive and behavior disorders in young children. It is worth noting that some intraoperative non-specific, patho-physiological changes may overlap with the effects of anesthetics. For example, exposure of the developing rodent brain to a high concentration of oxygen for a few hours during a specific period of development can also cause an apoptotic neurodegeneration in neurons from several major regions of the developing forebrain [154]. Thus, the effects of hyperoxia in perinatal medicine must be taken into consideration. In human studies, the effects of co-morbid condition and surgery on neurocognitive outcome are difficult to separate from effects of anesthetics alone. Other potentially confounding factors include intraoperative core body temperature [37]. Hypocapnia [155] may also play a role in pathogenesis of neuronal injury during clinical settings.

**Figure 1:**
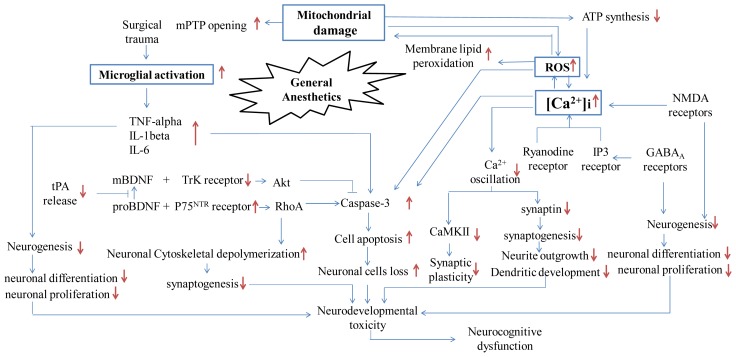
Schematic diagram presenting possible pathways by which general anesthetics induce early neurotoxicity in developing brain and later neurocognitive dysfunction. Note: ATP: Adenosine triphosphate; ROS: Reactive oxygen species; NMDA receptor: *N*-methy-d-aspartate receptor; GABAA receptor: Gamma-aminobutyric acid type A receptor; IP3 receptor: Inositol triphosphate receptor; mPTP: Mitochondrial permeability transition pore; TNF-alpha: Tumor necrosis factor-alpha; IL-1beta: Interleukin-1beta; IL-6: Interleukin-6; tPA: Tissue plasminogen activator; mBDNF: Mature brain-derived neurotrophic factor; proBDNF: Brain-derived proneurotrophic factor; Akt: Phosphorylated serine/threonine-specific protein kinase; CaMK II: Calcium/calmodulin-dependent Protein Kinase II; RhoA: Ras homolog gene family, member A; TrK receptor: Neurotrophic tyrosine kinase receptor; P75NTR receptor: Neurotrophin p75 receptor. ↑: increase; ↓: decrease; ⫞: block or inhibit.

Most studies have focused on the kind and duration of anesthetic exposure necessary to cause neurotoxicity, while recent studies have investigated how anesthetics can trigger neuronal apoptosis and degeneration, resulting in neurobehavioral deficiency. This change in research direction is timely, echoing Morgan and Sedensky’s statement, “We must move beyond studies that describe the magnitude and periods of vulnerability but leave us powerless to improve outcomes in exposed individuals, … and we are truly entering the next phase in the study of anesthetics and neurodegeneration.” [156]. Different types of anesthetic may have common mechanism(s) which lead to neurotoxicity. Here we reviewed the emerging molecular mechanisms on anesthetic-induced neurotoxicity (summarized in Figure 1) and possible neuroprotective strategies. Interestingly, most anesthetics (volatile anesthetics, ketamine or propofol) have dual effects on the developing brain; they can be neuroprotective and neurotoxic depending on the context. The details of this dual effect however, are still unknown. Furthermore, the effects of anesthetics on other aspects of the developing brain, such as activity-dependent neuronal plasticity, circuitry organization and functional connectivity, may be more complex. Therefore, we urgently need new studies that will further our understanding of developmental neurotoxicology, improve our ability to predict adverse effects in animals exposed to anesthesia, and assist in the extrapolation of these adverse neurological outcomes to risks to human health. Moreover, we need guidelines on how to prevent this neurotoxicity while not interfering with the beneficial actions of anesthetic drugs in perioperative medicine.
